# Evaluation of the safety and efficacy of suction‐tip forceps, a new tool for laparoscopic surgery, for gastric cancer

**DOI:** 10.1111/ases.12858

**Published:** 2020-09-10

**Authors:** Nobuyuki Sakurazawa, Jun‐ichiro Harada, Fumihiko Ando, Hiroki Arai, Komei Kuge, Satoshi Matsumoto, Youichi Kawano, Akihisa Matsuda, Hideyuki Suzuki, Hiroshi Yoshida

**Affiliations:** ^1^ Department of Surgery Nippon Medical School Chiba Hokusoh Hospital Chiba Japan; ^2^ Department of Digestive Surgery Nippon Medical School Tokyo Japan

**Keywords:** forceps, laparoscopic surgery, suction

## Abstract

**Introduction:**

Laparoscopic surgery is a minimally invasive surgery; however, obstacles to its functional optimization remain. Surgical ports can accommodate only one instrument at a time so complex exchange manipulations are necessary during surgery which increases operation times and patient risk. We developed a new laparoscopic instrument that functions as both forceps and a suction tube, which renders intraoperative tool exchange unnecessary. This pilot study was undertaken to evaluate the safety and efficacy of this novel dual‐function device in laparoscopic surgery for gastric cancer.

**Methods:**

This single‐center pilot study assessed patient safety during and after laparoscopic distal gastrectomy for gastric cancer with the suction‐forceps using intraoperative video and clinical follow‐up, respectively. To evaluate instrument efficacy, we measured the time interval between the start of any bleeding and the start of aspiration (“suction access time”) and compared this time with that of a conventional surgical setup.

**Results:**

In total 15 patients participated, with all procedures being successful. No excess tissue damage occurred during surgery. Suction access time was significantly shorter in cases of bleeding when the suction‐tip forceps were used for aspiration (2.01 seconds) compared to an ordinary suction tube (12.5 seconds; *P* < .01).

**Conclusion:**

These findings suggest that our new suction‐tip forceps are a useful, safe, and efficacious operative tool. This surgical innovation may considerably simplify gastric laparoscopic surgery. This pilot study was registered with Japan Clinical Trial Registration on 22 June 2017 (registration number: UMIN000027879).

## 1 INTRODUCTION

Since 1994, when Kitano et al first reported successful laparoscopy‐assisted gastrectomy,^1^ this technique has been widely adopted as a less invasive operative procedure for gastric cancer. However, in comparison with open surgery, laparoscopic surgery is more technically complex and tends to take more time.^2^, ^3^, ^4^, ^5^ One reason for this is that each surgical port in the abdominal cavity is able to accommodate only one surgical instrument at a time. When bleeding inevitably occurs during laparoscopic surgery, the surgeon must temporarily withdraw the ultrasonic laparoscopic coagulation shears (LCS) and substitute them with a suction tube for blood aspiration. This one‐handed substitution is somewhat complex and takes time. If a substitution period takes too long, small intraoperative bleeding is likely to penetrate the surrounding tissue, which discolors the surrounding tissues with a reddish hue making identification of dissection layers difficult even after blood aspiration.^6^ To address this limitation, there is one surgical tool, composed of the conventional forceps (inserted through an incision) and connected with an external suction tube, developed for use in uniportal video‐assisted thoracoscopic surgery.^7^ However, that tool could not be inserted through a 5‐mm diameter port, which is standard in laparoscopic surgery; furthermore, it is not designed for free rotation.

To better address the practical challenges of laparoscopic surgery, we developed a novel type of forceps inlaid with a suction‐tip for simultaneous aspiration. During surgery, the surgeon can use these suction‐tip forceps with one hand to begin aspiration as soon as bleeding occurs and, without changing instruments, use the LCS in the other hand to coagulate the origin of bleeding. Our instrument is the first of its kind to be narrow enough for insertion into the standard 5‐mm port used in laparoscopic surgery, to function as both forceps and as a suction device, and to have 360° rotational capacities. This single‐center pilot study was undertaken to evaluate the safety and efficacy of this tool in a real‐world setting. Forceps and suction tubes for laparoscopic surgery are very basic tools that have demonstrated safety that is widely recognized. Therefore, our suction‐tip forceps, which combines these two treatment modalities, was considered safe for human research and was approved by our institution's ethics committee.

## 2 MATERIALS AND METHODS

Between June 2017 and April 2018, 15 patients with gastric cancer underwent laparoscopic surgery with the suction‐tip forceps at Nippon Medical School Chiba Hokusoh Hospital, Chiba, Japan.

Due to the fact that this was a feasibility study, power analysis to calculate sample size was not necessary. The study protocol was reviewed and approved by the institutional review board of Nippon Medical School Chiba Hokusoh Hospital (Ethics Committee of Nippon Medical School Chiba Hokusoh Hospital, Approval No. 583). All patients were older than 20 years, were not suitable candidates for endoscopic mucosal resection or endoscopic submucosal dissection, and were judged to be candidates for laparoscopic treatment. Participants provided written informed consent prior to surgery. Additionally, this study was registered at Japan Clinical Trial Registration database (registration number: UMIN000027879) with the title: Safety Examination of Endoscopic Surgical Forceps which can Absorb at the Tip. It adheres to the Consolidated Standards of Reporting Trials 2010 guidelines appropriate for non‐randomized pilot studies.

This study was carried out in compliance with the Declaration of Helsinki. The protocol was reviewed and approved by the institutional review board of Nippon Medical School Chiba Hokusoh Hospital, and written informed consent was obtained from each patient before his or her participation in the study (Ethics Committee of Nippon Medical School Chiba Hokusoh Hospital, Approval No. 583).

**FIGURE 1:**
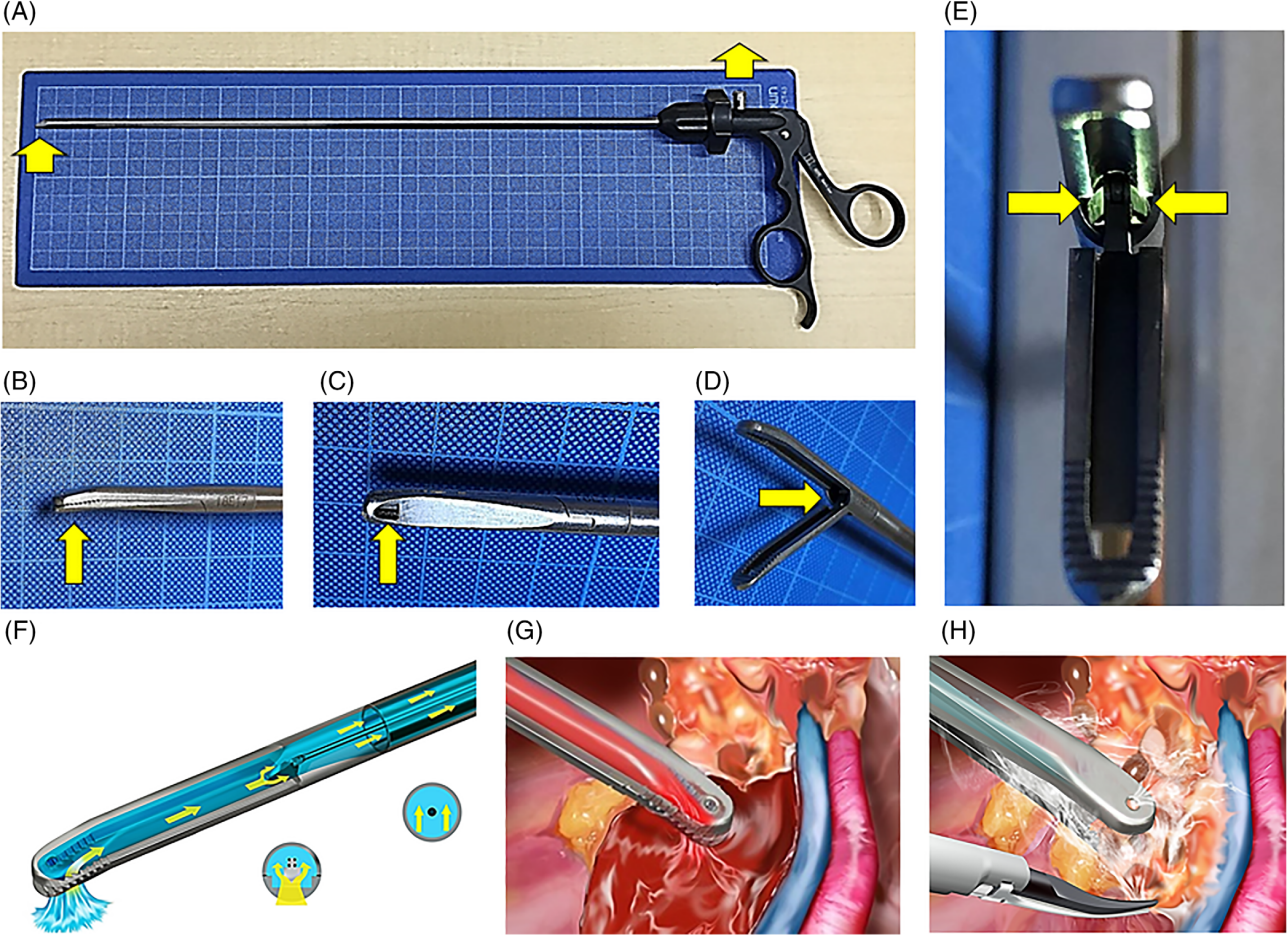
Design features of the suction‐tip forceps. The yellow arrow denotes suction flow in all panels. A, Suction‐tip forceps. B, Side view of the closed jaws. C, Suction hole positioned at the tip of lower jaw. D, Oblique view of the opened jaws. E, Front view of the bilateral canal within the pivot joint. F, Oblique transparent view of the suction flow in closed jaws. G, Blood suction at the tip of closed jaws. H, Mist suction from gap in semi‐opened jaws while tissue is grasped

The suction‐tip forceps can be smoothly inserted and withdrawn through ordinary 5‐mm surgical ports and it has an inlaid 2.5‐mm suction tunnel, and thus are capable of aspirating matter from the tip of the forceps' jaws to the handle (Figure 1A,F) for evacuation via a vacuum system. If the jaws are closed, a 2.5‐mm diameter tunnel is formed between the jaws (Figure 1B,C,F), and blood can be aspirated from this tip (Figure 1B,C,G). If the jaws are open, surgical mist can be aspirated from the pivot joint to the handle (Figure 1D,E,H). The suction‐tip forceps (Dolphin suction‐tip forceps, Hope Denshi Co. Ltd., Kamagaya, Chiba, Japan) have been commercially available in Japan as of 23 January 2018.

**FIGURE 2:**
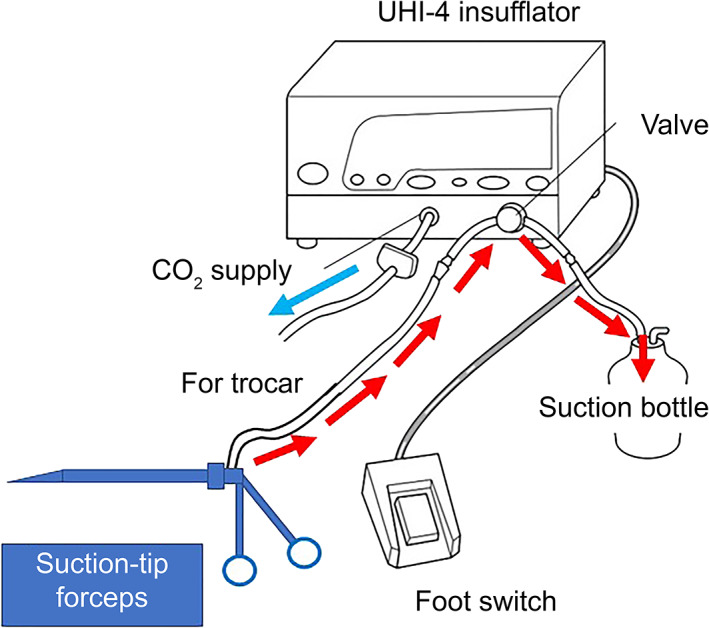
Foot‐controlled suction system used during surgery. Illustration of the UHI‐4 system (Olympus Medical Systems, Tokyo), modified from that of Shinsuke et al^8^

**FIGURE 3:**
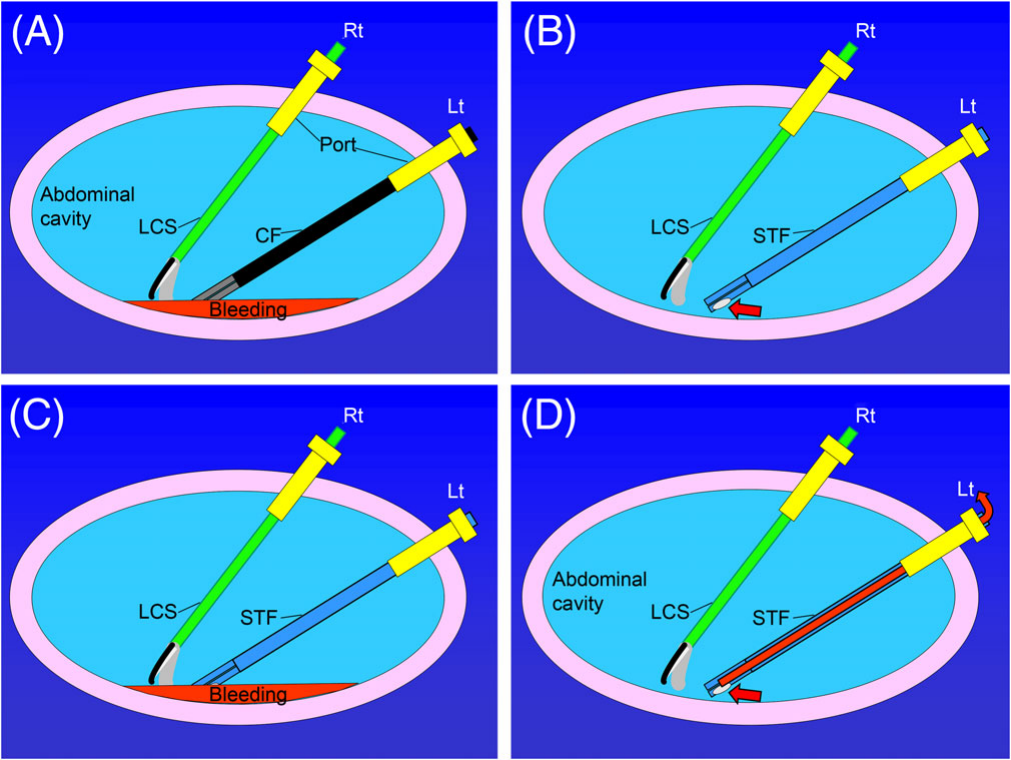
Overview of suction system. A, Conventional positioning of forceps during bleeding. B, Positioning of suction‐tip forceps (red arrow points to suction hole at the tip). C, Positioning of suction‐tip forceps before bleeding. D, Positioning of suction‐tip forceps after suction of bleeding, with dry area (red arrow, evacuation flow of bleeding). CF, conventional forceps; LCS, laparoscopic coagulation shears; Lt: operator's left hand; Rt: operator's right hand; STF, suction‐tip forceps

A UHI‐4 device (Olympus Medical Systems, Tokyo) was used to perform abdominal insufflation. This device is designed to provide CO_2_ when the foot pedal is pushed in the event of mist or smoke discharge in the surgical field. In our setup (modified from Shinsuke et al^8^) one end of the discharge tube was connected to a negatively pressurized suction bottle and the other end was connected to an outlet above the handle of the suction‐tip forceps. In this way, we created a system by which negative pressure could be applied to the suction‐tip forceps when the foot pedal is pushed, which allows aspiration of mist or smoke, as well as blood, through the tip of the forceps (Figure 2). A conceptual schematic of suction‐tip forceps use is shown in Figure 3.

All surgical procedures were conducted by three surgical specialists (NS, JH, and FA). Patients were placed in the supine position with their legs apart after induction of general anesthesia. The pneumoperitoneum was inflated with an UHI‐4 insufflator (Olympus Medical Systems) at 8 to 12 mm Hg. Five ports were then made in a reverse trapezoid shape on the upper abdominal side of the patient. The surgical operator stood to the right side of the patient, controlling LCS with the right hand and the suction‐tip forceps with the left hand while watching the laparoscope's Video S1 feed on a screen positioned above the patient. An assistant surgeon stood to the left side of the patient, holding conventional forceps in each hand. A scopist stood between the patient's legs, controlling the laparoscope inside the body cavity.

In the event of bleeding, blood was aspirated with suction‐tip forceps held in the operator's left hand. Simultaneously, the operator used the right hand to establish hemostasis with cauterization via the LCS. While the suction‐tip forceps were used by default during surgeries, in cases of massive bleeding or other complications, the operator occasionally withdrew the LCS (with the right hand) and replaced it with an ordinary suction tube that had a monopolar soft coagulation tip for hemostasis.

Our intraoperative approach was as follows: gastrocolic ligaments were dissected with the LCS so as to enter the omental bursa. The left gastroepiploic artery and vein were then identified, clipped, and dissected. Next, the right gastroepiploic vein and artery were identified, clipped, and dissected. The duodenum was dissected with a stapler. After that, the right gastric artery and vein were identified, clipped, and dissected. The 8a lymph nodes along the common hepatic artery were then excised. Next, the left gastric artery was double‐clipped and dissected. The stomach was dissected with a stapler (distal gastrectomy). This wound in the umbilical region was dilated to 4 cm, and specimens were removed. The residual portion of the stomach was anastomosed to the duodenum (Billroth‐I, delta anastomosis). Finally, after washing of the surgical cavity and confirmation of hemostasis, a drain was inserted into the anastomosed area, and the abdomen was closed.

To evaluate the clinical safety of the new suction‐tip forceps, outside experts (HA, KK, SM, and YK) independently reviewed the surgical Video S1 to detect any unnoticed damage that may have occurred during use (e.g., tearing or piercing of tissue); they did not attend any surgeries in person. Postoperative complications were evaluated according to the Clavien‐Dindo classification.^9^, ^10^

To evaluate the efficacy of the suction‐tip forceps, we assessed suction access time, defined as the time interval between the start of bleeding and the start of aspiration. We compared suction access time as when the suction‐tip forceps were used with that of the conventional substitution technique (withdrawal of the LCS with the surgeon's right hand, switching to an ordinary suction tube, and reinsertion into the source of bleeding). Use of the suction‐tip forceps was the default method during surgery; the conventional method was used in certain situations as judged by the operator (e.g., when LCS alone could not stop bleeding properly and extra suction stabilization was required). In other words, when the operator determined that hemostasis due to soft coagulation was appropriate, he used a normal suction tube (having a coagulation function at the tip) and stopped bleeding by soft coagulation. From data analysis, we excluded cases in which both suction methods were used for one bleeding instance.

Statistical analysis was performed with the R software suite (version 3.3.2; The R Foundation for Statistical Computing, Vienna, Austria). The distribution of data was not normally distributed as determined by Kolmogorov‐Smirnov test, so we assessed the data by using the Mann‐Whitney test. A *P* value of >.05 was regarded as indicative of statistical significance.

## 3 RESULTS

Table 1 lists the characteristics and surgical outcomes of 15 patients with gastric cancer who underwent laparoscopic distal gastrectomy in this trial. The mean age of the patients was 72.5 years, the mean length of surgery was 244.3 minutes, and the mean amount of blood loss was 18.0 g. The length of hospitalization after the surgery had a mean of 12.9 days and the mean length of follow‐up after discharge was 17.3 months. Histopathological and radiological evaluation of the tumors revealed stage IA cancer in nine cases, stage IB in four cases, and stage IIB in two cases. In all cases, R0 surgery was successful (Table 1).

**TABLE 1:** Patient's characteristics and surgical outcomes

		Cancer features					Operation					Suctioning equipment	
Case no.	Gender	Location in gastrointestinal tract	Circle	Macro type	Histo type	BMI (kg/m^2^)	Time (min)	Blood loss (g)	Hospital stay (d)	Final diagnosis of stage	Follow‐up (mo)	Suction‐tip forceps	Ordinary suction tube
1	Female	Lower	Lesser curvature	IIa + IIc	Por2	21.7	220	10	9	IB	22	7	3
2	Male	Middle	Lesser curvature	IIc	Tub1	24.7	302	10	13	IA	20	8	4
3	Male	Middle	Posterior wall	III	Por1	21	267	10	10	IB	16	8	3
4	Male	Lower	Greater curvature	IIc	Por2	24.2	250	35	12	IB	21	9	5
5	Male	Lower	Lesser curvature	IIa + IIb	Tub1	30.9	271	40	24	IA	12	7	4
6	Male	Lower	Anterior wall	IIc	Tub1	23.6	158	10	14	IA	18	7	2
7	Female	Lower	Anterior wall	IIc	Tub2	28.9	239	10	12	IA	20	4	5
8	Female	Middle	Greater curvature	IIa + IIc	Por2	30.7	273	10	16	IA	19	14	7
9	Male	Lower	Lesser curvature	IIa + IIc	Tub2	23.8	260	75	12	IIB	17	6	4
10	Female	Lower	Lesser curvature	IIa + IIc	Por2	19.2	268	10	16	IA	18	10	1
11	Female	Lower	Lesser curvature	IIa + IIc	Tub1	21.1	223	10	13	IA	20	7	0
12	Female	Lower	Anterior wall	IIc	Tub2	24.5	198	10	14	IA	17	12	2
13	Male	Lower	Lesser curvature	3	Tub2	18.9	259	10	10	IIB	14	7	2
14	Male	Lower	Lesser curvature	IIc + III	Sig	25.7	289	10	10	IB	13	4	3
15	Female	Lower	Greater curvature	IIc	Sig	20.8	188	10	8	IA	13	10	2
Avg.						24.0	244.3	18.0	12.9		17.3	8.0	3.1

*Note*: Patient age range: 43 to 90 years (average 72.5). Macro type: stage according to macroscopic appearance; Por 1: poorly differentiated adenocarcinoma (solid type); Por 2: poorly differentiated adenocarcinoma (non‐solid type); Sig: signet ring cell carcinoma; Tub 1: well‐differentiated tubular adenocarcinoma. Tub 2: moderately differentiated tubular adenocarcinoma. Histo type: histological type. When used gauzes were dry and aspiration bottle was empty, final blood loss was estimated as 10 g to account for blood remaining in the tube connected to the suction bottle.

Only one non‐related post‐surgical complication occurred out of 15 surgical cases; patient no. 5 developed a Clavien‐Dindo postoperative complication (grade 3). This patient had a history of previous heart failure with angina pectoris, and postoperative computed tomography showed no abnormalities such as pancreatic fistula or leakage in the abdomen after surgery. It was concluded that this complication was diagnosed as pleural effusion caused by worsening of heart function, not by intra‐abdominal surgery. It was alleviated with thoracic drainage. This patient later died of heart failure 12 months after surgery. Another case, patient no. 3 died from cerebral hemorrhage 16 months after surgery; the remaining 13 patients remained stable without disease recurrence through June 2019.

There was no tissue damage observed by the suction‐tip forceps according to independent Video S1.

**FIGURE 4:**
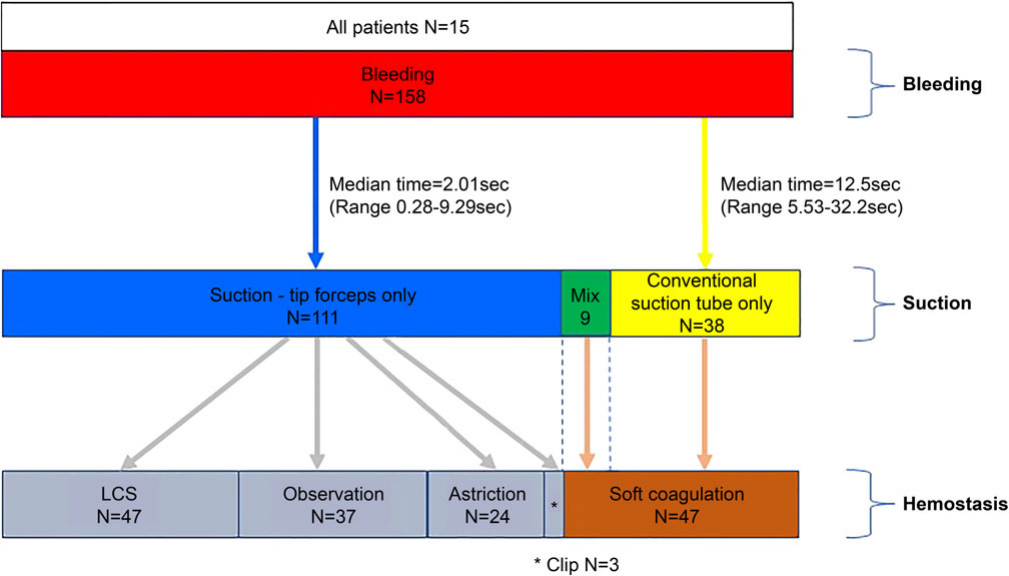
Results of different approaches to controlling bleeding

There was a combined total of 158 bleeding instances among 15 patients. As seen in Figure 4, these instances of bleeding were divided into 111 in which only the suction‐tip forceps were used, 38 in which only substitution with an ordinary suction tube was used, and nine in which both methods were used (not included in analysis). Suction access time with the suction‐tip forceps was compared with the suction access time for the conventional method (replacement of the LCS with an ordinary suction tube). The medians were 2.01 seconds (range: 0.28 to 9.29 seconds) and 12.5 seconds (range: 5.53 to 32.2 seconds), respectively (*P* < .01).

**FIGURE 5:**
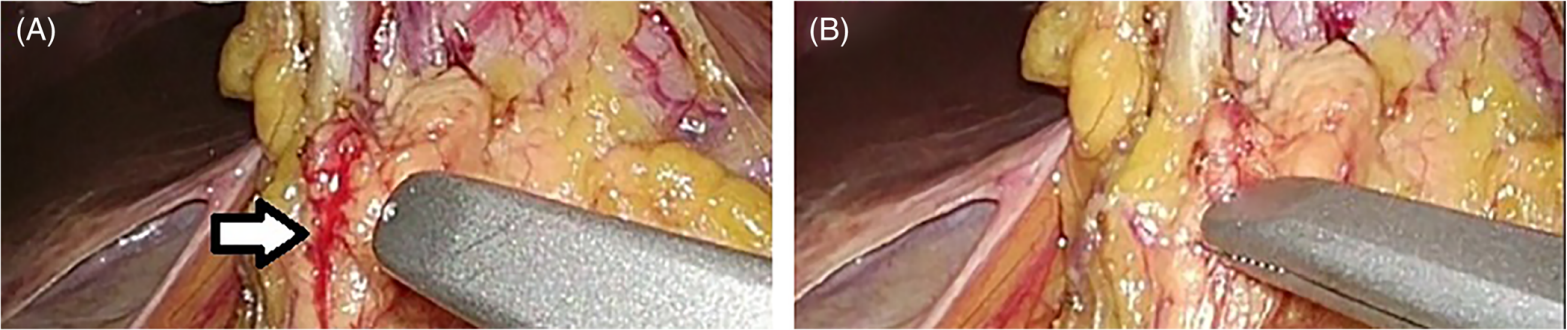
Suction of small bleeding. A, Before suction; white arrow shows bleedings of surgical site. B, After suction

**FIGURE 6:**
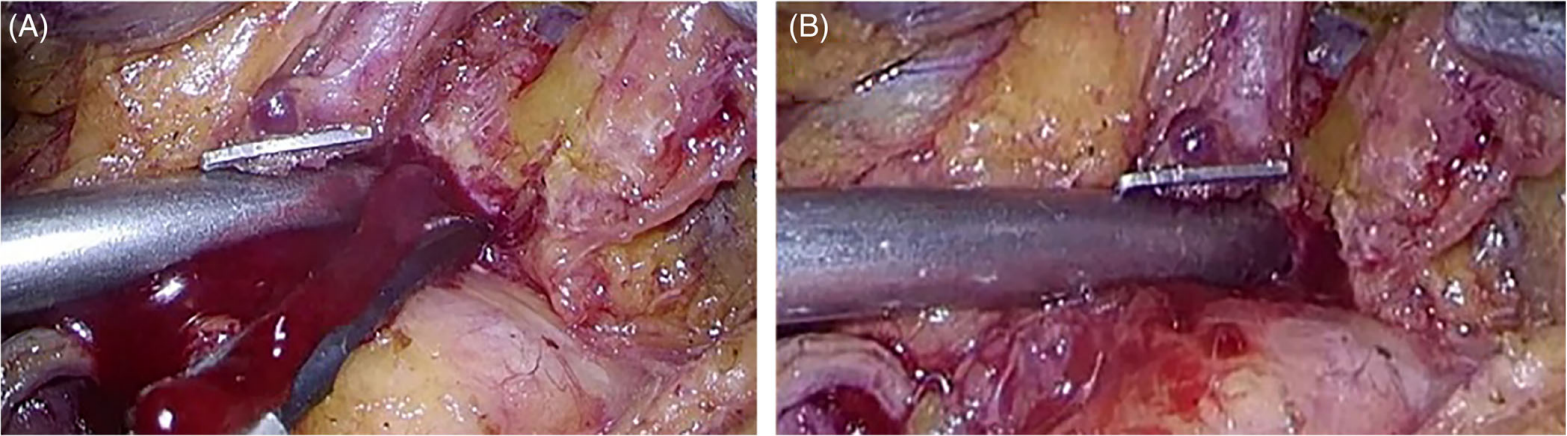
Suction of massive bleeding. A, Before suction of massive bleeding. B, After suction of massive bleeding

For hemostasis of the 158 bleeding instances, aspiration was performed with the suction‐tip forceps without any further manipulation (no further bleeding after aspiration) in 37 such instances; hemostasis was achieved with LCS use soon after aspiration with the suction‐tip forceps in 47 instances; compressive hemostasis was needed after aspiration with the suction‐tip forceps in 24 instances; clipping was necessary in three instances; and the suction‐tip forceps required follow‐up treatment with an ordinary suction tube to achieve hemostasis in 47 instances (Figure 4). The suction‐tip forceps could aspirate volumes of blood ranging from small to massive (Figures 5 and 6).

**FIGURE 7:**
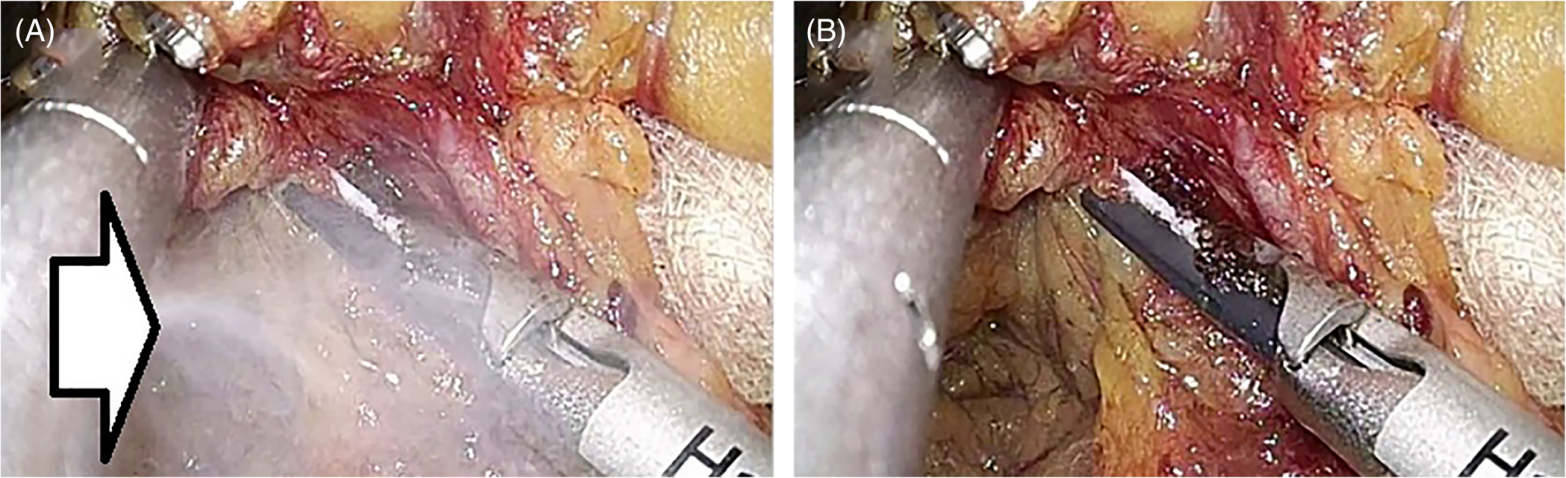
Suction of mist. A, Before suction of mist; white arrow shows mist generated by laparoscopic coagulation shears. B, After suction of mist by suction‐tip forceps

Qualitatively, the suction‐tip forceps quickly and effectively evacuated the surgical mist created by the LCS in the surgical field many times, according to Video S1 analysis (Figure 7).

## 4 DISCUSSION

This is the first report of a new surgical forceps design. This novel tool has a tip that is capable of intraoperative aspiration, can be passed through a 5‐mm port and can be rotated 360° without restriction.

Our analysis showed that the use of this type of forceps in laparoscopic gastrectomy was technically feasible and R0 surgery was accomplished in all 15 cases. No complications or tissue damage associated with the use of the suction‐tip forceps were observed in any patient.

In laparoscopic gastrectomy, five ports are usually employed; the operator holds the LCS with the right hand and the forceps with left hand, the assistant holds the forceps with both hands, and the scopist holds a laparoscope at the umbilical port.^11^ When aspiration is needed to address bleeding or exudate, the operator usually withdraws the LCS and inserts a suction tube via a port. The suction‐tip forceps designed by our team has an inlaid suction tube. This allows for immediate aspiration upon detection of bleeding (median time: 2.01 seconds; number of instances of bleeding: 111; Figure 4), without the need for substitution with another suction tool. In the conventional surgical technique (replacement of the LCS in the right hand with an ordinary suction tube upon the detection of bleeding) mandated a median of 12.5 seconds before aspiration could be started (in 38 cases; Figure 4). The suction‐tip type of forceps allowed aspiration to be started more rapidly after the onset of bleeding and also reduced the complexity of intra‐surgical manipulations; namely, replacement of the LCS held in the right hand with a suction tube. Without the need to perform a complex instrument substitution, operational stress is significantly reduced as well.

The measures taken after bleeding are shown in Figure 4. There were 37 additional instances of bleeding that were minor or in which the blood pool was very small and did not necessitate hemostasis after aspiration. In the case of sudden massive bleeding, a surgeon was able to begin aspiration with the left hand as quickly as 0.28 seconds with the suction‐tip forceps after detection of bleeding. The operator was able to address bleeding with suction‐tip forceps in 111 (70.3%) of 158 instances of intraoperative bleeding without the need for coagulation with an ordinary suction tube. The remaining 47 instances of intraoperative bleedings were addressed with an ordinary suction tube that we used with a monopolar soft coagulation at the tip,^12^, ^13^, ^14^ because the suction‐tip forceps did not have a coagulation function. Therefore, the forceps can only aspirate bleeding. However in the next model, we are considering a coagulation function.

The mist and smoke (mixed) generated from the LCS and moisture during surgery can obscure the surgical field of vision.^15^, ^16^ Surgical smoke has been reported to be hazardous to physicians, nurses, and other medical staff involved in the operation if it escapes from the patient's body,^17^, ^18^ but it also has adverse effects on the patient, if it spreads within the peritoneal cavity as mist.^19^ We observed that the suction‐tip forceps aspirated any surgical gases created by moisture in the intraoperative field many times during video analysis. This evidence suggests that the suction‐tip forceps can aspirate both gas (mist in Figure 7) and liquid (blood and leachate in Figures 5 and 6), thereby consistently maintaining a clear surgical field.

During conventional laparoscopic surgery, mist that has spread within the peritoneal cavity is ventilated with a smoke extraction apparatus. The surgical ports are the point of evacuation but only after the mist has spread from the tip of LCS and obstructed the surgical field. Mist can be aspirated more efficiently if the aspiration is performed close to the point of origin of the mist before it diffuses within the peritoneal cavity.^20^, ^21^ Aspiration of intraoperative mist with the suction‐tip forceps is advantageous in terms of the proximity to the origin of the mist. The distance from the LCS's tip to port (conventional evacuation setting) is far longer than that for the suction‐tip forceps tip nearby in the surgical field. If the suction‐tip forceps are held with the left hand, the mist and smoke generated from the LCS held with the right hand can be aspirated at a very short distance, thus efficiently improving the visual field, and absorption of the surgical smoke may be more efficient.

Limitations of this study were that it was conducted at a single center and was not a randomized controlled trial. In addition, the number of treated patients and instances of bleeding were small, which limited statistical power. Finally, the assessment of surgical mist cleared by our tool remains anecdotal.

Nevertheless, our newly developed suction‐tip forceps have dual functions as forceps and a suction tube that were shown to be safe for use in gastric laparoscopic surgery. This innovative tool can grasp tissue, as well as aspirate blood, mist, and smoke, without the need for substitution or supplementary instrumentation. This surgical innovation reduces operator burden and improves the agility with which the surgeon can respond to intraoperative bleeding. Future high‐powered trials should be conducted to quantify the effect of suction‐tip forceps on different types of laparoscopic surgery.

## Supporting information


https://drive.google.com/file/d/1AkFBzyFdRPeeMBzU8lNbDQQqjOvQwZFh/view
**Video S1** Scene 1: A common case where the lymph node along the splenic artery bled during dissection. Suction was performed with the suction‐tip forceps without a time lag to maintain the visual field. After coagulation and hemostasis, there was more bleeding, but was again immediately suctioned to keep the surgical field clear.
**Scene 2:** A rare case of massive bleeding caused by laparoscopic coagulation shears (LCS) cavitation during lymph node dissection along the portal vein. As the forceps have suction ability up to 300 mL of blood per minute, the surgical field was immediately cleared to maintain a clear field of view. After controlling bleeding and pressing with gauze to completely stop bleeding.
**Scene 3:** During lymph node dissection along the right gastric omental vein, mist appeared after LCS application on fat tissue. However, the mist was quickly aspirated and a clear field of view was maintained.
